# Royal Medical and Chirurgical Society

**Published:** 1897-06-12

**Authors:** 


### Medical Progress and Hospital Clinics.

[*The Editor will be glad to receive offers of co-operation and contributions from members of the profession. All letters should be addressed to* The Editor, at the Office, 28 & 29, Southampton Street, Strand, London, W.C.]

### ROYAL MEDICAL AND CHIRURGICAL SOCIETY.

A paper was read at the last meeting held on June 8th by Dr. W. Howship Dickinson on the Occurrence of Musical Mitral Murmurs in Connection with Aortic Stenosis.

Four cases were related in which a musical murmur, thought to be produced by mitral regurgitation, was associated with obstruction of the aortic exit. In one the mitral valve was practically sound, in the others the mitral valve was diseased as well as the aortic.

Case 1 was that of a boy who had, together with much evidence of intra-ventricular tension, an intense apex systolic murmur which had a musical tone. After death the aortic orifice was found to be almost completely closed, the mitral valve practically natural. The mitral murmur, as it was thought to be, was the only murmur heard. It was attributed to the over-distension of the ventricle due to the obstruction of its aortic exit, and to consequent leakage of the mitral valve under the abnormal pressure to which it was subjected.

Case 2 was that of a man who, after having been for some time under treatment with the ordinary signs and symptoms of disease of the aortic valve, developed a murmur indicative of mitral regurgitation, which was attended with a definite squeak like that of a mouse. After death the heart was found to be greatly hypertrophied, especially as to the left ventricle. The pericardial cavity was obliterated by old adhesions. The aortic orifice was obstructed by fibroid thickening, the mitral crescentic and contracted.

Case 3 was that of an old man who displayed shortly after admission, and shortly before his death, a systolic whistle in the mitral area. The left ventricle was found to be hypertrophied, the aortic valves calcified and the opening contracted; the mitral valve thickened.

Case 4 was that of a man 54 years of age, who had at the apex a distinct systolic squeak like the high note of a fiddle. The heart was found to be hypertrophied. The aortic orifice was almost closed, partly by old rigid thickening, partly by recent soft vegetations. The edges of the mitral valve were slightly thickened, as also were those of the tricuspid. The musical murmur was attributed, as in the other cases, to the occurrence of mitral regurgitation in small volume and at high pressure, the high pressure being due to the ventricular hypertrophy and the aortic obstruction.

The cases recorded were held to indicate that in aortic obstruction, together with hypertrophy of the left ventricle, a peculiar tone may be imparted to mitral regurgitation, probably in consequence of the force under which it is produced.

